# Stromal cell‐derived factor‐1 regulates the secretion of interleukin‐1β in the temporomandibular joint of rats with synovial inflammation

**DOI:** 10.1111/jop.13040

**Published:** 2020-06-13

**Authors:** Dong Qi, Shuzhen Sun, Liang Han, Qiang Wang, Jingjing Kong, Yujun Zhang, Ronglin Wang, Yan Chen, Ping Ji

**Affiliations:** ^1^ Department of Prosthodontics Shandong Key Laboratory of Oral Tissue Regeneration & Shandong Engineering Laboratory for Dental Materials and Oral Tissue Regeneration Shandong University Stomatology Hospital Jinan China; ^2^ Department of Prosthodontics Jinan Stomatological Hospital Jinan China

**Keywords:** AMD3100, CXCR4, IL‐1β, occlusal interference model, SDF‐1, synovitis, temporomandibular joint

## Abstract

**Background:**

Synovitis is characterized by the infiltration of inflammatory cells and often accompanies the pathological progression of the clinical symptoms affecting the temporomandibular joint (TMJ), such as pain, snapping, and limited mouth opening. It has been suggested that the signal transduction pathway and resultant proinflammatory mediators play important roles in the pathogenesis of synovitis. Therefore, in this present research, we aimed to investigate the changes in the expressions of stromal cell‐derived factor 1 (SDF‐1) and interleukin (IL)‐1β in rats with occlusal interference.

**Materials and Methods:**

We divided 36 male Wistar rats into the following groups: Group A (control group), Group B (occlusal interference group), and Group C (AMD3100 group). Synovial inflammation was induced in the rats in Groups B and C to establish the occlusal interference model. The inflammatory changes were detected, and the expressions of SDF‐1 and IL‐1β in the synovium were assayed via immunostaining and a real‐time quantitative polymerase chain reaction (PCR).

**Results:**

In Group B, obvious inflammatory changes were observed in the synovial membranes; additionally, the SDF‐1 and IL‐1β expression levels were significantly higher at the protein and mRNA levels. However, in Group C, these experimental results were inhibited by an injection with AMD3100.

**Conclusion:**

These results may indicate that SDF‐1 regulates the expression level of inflammatory factors, such as IL‐1β, in the synovial membranes of rats with occlusal interference. Our findings suggest that the SDF‐1 axis may contribute to the onset of synovitis during the development of TMJ joint disease.

## 1 INTRODUCTION

The temporomandibular joint (TMJ) is one of the most intricate and complicated joints in the body. It is one of the important organs in oral and maxillofacial region, and it participates in a series of facial movements, such as chewing, swallowing, forming facial expressions, and pronouncing. The synovial membrane that secretes synovial fluid is located on the inner side of the joint capsule and is considered to be a sensitive tissue in the articular cavity that experiences stress. The synovial fibroblasts of the TMJ are believed to play important roles in the progression of synovitis.

Synovitis is characterized by the infiltration of inflammatory cells and often accompanies the pathological progression of the clinical symptoms that affect the TMJ, such as pain, snapping, and limited mouth opening. The inflammatory changes in the TMJ synovial lining of patients with temporomandibular disorders (TMD), such as internal derangement (ID) or osteoarthritis (OA), are investigated by conducting arthroscopical or histopathological analyses.^1^ Additionally, mounting evidence suggests that the resulting proinflammatory mediators, such as interleukin (IL)‐1β, tumor necrosis factor‐α (TNF‐α), and matrix metalloproteinases (MMPs), play important roles in the pathogenesis of synovitis and OA as they affect the articular cartilage.^2^, ^3^ Therefore, the timely prevention and reduction of the release of the corresponding inflammatory factors are of great significance.

IL‐1β is a proinflammatory cytokine that is involved in a variety of pathological processes, such as tissue destruction and edema formation. Some researchers have pointed out that the high expression of inflammatory factors, such as IL‐1β, has considerable effects on the synovial inflammatory response and on cartilage destruction.^4^, ^5^ Stromal cell‐derived factor 1 (SDF‐1), also known as CXCL‐12, is a CXC chemokine that elicits cell chemotaxis. Current studies have found that SDF‐1 has two receptors, CXC chemokine receptor 4 (CXCR4) and CXCR7.^6^, ^7^ It has been suggested that the expression of SDF‐1 is abnormally increased in the articular cavity of patients with synovitis or OA.^8^

The SDF‐1/CXCR4 signaling pathways have attracted the attention of many scholars. In this study, we induced TMJ synovial damage in rats to establish an occlusal interference rat model in order to learn more about the SDF‐1/CXCR4 signaling pathways. The aim of this study was to investigate the changes in the levels of SDF‐1 and IL‐1β in the synovial membrane of the TMJ in rats following the administration of AMD3100. AMD3100 is a non‐peptide bicyclam and is able to specifically antagonize the CXCR4 receptor at three main interaction residues located around the main ligand binding pocket of CXCR4 in transmembrane domains IV, VI, and VII. The binding of AMD3100 competitively inhibits the binding of CXCL12 and prevents subsequent downstream signaling.^9^ This study may provide new theoretical evidence for the pathologic mechanism of synovial inflammation in the TMJ.

## 2 MATERIALS AND METHODS

### 2.1 Animal preparation

We used 36 male rats (Wistar, 6 weeks old, weighing 160‐180 g, obtained from the Experimental Animal Center of Shandong University, China) that had no oral and maxillofacial dysfunctions. They were housed in the laboratory at a temperature of 25°C and relative humidity of 55%, and a 12‐hour light/dark cycle was maintained; further, they could obtain food and water at will. Forty rats were approved for use in this research by the animal care and use committee of Shandong University.

### 2.2 Grouping and modeling

The rats were randomly divided into three groups: (a) Group A (control group), (b) Group B (occlusal interference group), and (c) Group C (AMD3100 group). The rats in Group B and Group C were anesthetized via the administration of an intraperitoneal injection of pentobarbital sodium (0.5%, 40 mg/kg), and a metallic crown (0.6 mm, uniform thickness) was bonded onto the first right mandibular molar to establish the occlusal interference model.^10^ Contrarily, the rats in Group A were anesthetized, and their mouths were forced open for approximately 5 minutes using a protocol similar to the one used in the occlusal interference groups; however, no crowns were bonded. The rats in Group A and Group B were injected with saline solution into the upper joint cavities of both sides of the TMJ (20 µg diluted in 40 µL dimethyl sulfoxide (DMSO) on day 1, 5, 8, and 12), and those in Group C were injected into the upper joint cavities of both sides of the TMJ with AMD3100 (20 µg diluted in 40 µL DMSO on day 1, 5, 8, and 12; Selleck Chemicals, USA). The entire experiment lasted for 2 weeks.

### 2.3 Tissue preparation

After 2 weeks, each group was randomly divided into two subgroups (6 rats per subgroup). Six rats were used for hematoxylin and eosin (HE) staining and an immunohistochemical analysis, and the others were used for a real‐time polymerase chain reaction (PCR). All the animals were deeply anesthetized and were transcranially perfused with 4% paraformaldehyde in phosphate buffer (0.1 mol/L, pH = 7.4). Subsequently, the rats were perfused with heparinized saline, followed by a cold fixative containing 4% paraformaldehyde in 0.01 mol/L phosphate buffer saline (PBS, pH 7.2) for HE staining and immunohistochemistry tests. The tissue from the right side of the TMJ was fixed in 4% paraformaldehyde, clipped, demineralized, dehydrated, embedded, and cut along the sagittal plane into slices with a thickness of 4 µm. The TMJ synovial tissues on the right side of the other rats were collected for the PCR, and they were stored at −80°C.

### 2.4 Histological analysis

TMJ sections were randomly selected and stained with HE. Digital images were captured using a microscopic digital camera system (total magnification, 400X; Olympus). The severity of synovial tissue damage was evaluated using infrapatellar fat pad (IFP) inflammation scores on a scale consisting of 0‐3 points.^11^, ^12^

1. Dilated vasculature was graded on a scale with scores ranging from 0 to 3: grade 0, not present; grade 1, involving less than one‐third of the synovial membrane length; grade 2, involving one‐third to two‐thirds of the synovial membrane length; and grade 3, involving more than two‐thirds of the synovial membrane length.
2. Synovial lining hyperplasia was graded on a scale with scores ranging from 0 to 2: grade 0, staining of 1‐3 layers; grade 1, staining of 4‐6 layers; and grade 2, staining of 7 or more layers.
3. Vascularity was graded on a scale with scores ranging from 0 to 2: grade 0, a limited number (less than 5) of blood vessel profiles/mm^2^; grade 1, the focal occurrence of 5‐10 small blood vessel profiles/mm^2^; and grade 2, the focal occurrence of a large number (more than 10) of small blood vessel profiles/mm^2^.
4. Fibrin deposits were graded on a scale with scores ranging from 0 to 3 (the grades are similar to those described for the vasculature).

### 2.5 Immunostaining

According to the manufacturer's recommendations, in all the groups, the expressions of SDF‐1 and IL‐1β in the paraffin sections were examined using an indirect immunohistochemical approach. First, the tissue antigens were repaired using 0.125% trypsin in a 37°C incubator for 20 min, following which the experimental sections were incubated in goat serum and were then incubated overnight at 4°C with the primary antibodies against IL‐1β (1:1000, Cell Signaling) and SDF‐1 (bs‐4938R, rabbit polyclonal, Bioss, USA, 1:1300 dilution), respectively. Subsequently, we used a secondary antibody kit (Zhongshan‐Golden‐Bridge‐Biotechnology). Following this, they were exposed to a solution of horseradish peroxidase‐conjugated avidin‐biotin complex (ZSGB‐Bio, Beijing, China) for 20 min at 37°C. Next, the sections were visualized with 0.1% DAB (3, 30‐diaminobenzidine dihydrochloride) (ZSGB‐Bio) and were counterstained with hematoxylin. We used a microscopy‐digital camera system (Olympus) to acquire digital images. The immunostaining of SDF‐1 and IL‐1β in each type of the TMJ cells was semiquantitatively evaluated using the Image‐Pro Plus 6.0 software. The mean of the mean optical density (MOD) of three sections was considered as the relative protein expression in each rat.

### 2.6 Real‐time quantitative PCR

Total RNA was isolated from the synovial tissues using the TRIzol reagent (Invitrogen) according to the instructions. The synovial tissues were ground into a powder with liquid nitrogen using a mortar. The reverse transcription was carried out using the Prime Script TM RT reagent Kit (Takara) with 10 μL of gDNA Eraser in a reaction volume containing 1 μL of total RNA. The real‐time PCR was performed using SYBR® Premix Ex Taq ™ II (Tli RNaseH Plus) (TAKARA) and a 7500 real‐time PCR System. The target genes were normalized against the reference genes, such as the glyceraldehyde 3‐phosphate dehydrogenase (GAPDH) gene, and were quantified by determining the cycle threshold value (CT). Relative quantitation of target gene expression was achieved by employing the comparative CT method. The following primers were used: for SDF‐1:5′‐CCTGCCGATTCTTTGAGAGC‐3′ and 5′‐GTTGTTGCTTTTCAGCCTTGC‐3′ and for IL‐1β: 5′‐ACAAGGAGAGACAAGCAACGA‐3′ and 5′‐TCTGCTTGAGAGGTGCTGATG‐3′. The mRNA expression in each sample was determined three times, and the average value of the results of three repeated trials was used.

### 2.7 Statistical analysis

We used means ± SD to record normally distributed variables. The data in the different groups were compared by performing one‐way analysis of variance comparison tests, followed by least significant difference comparison tests. The results were analyzed using the SPSS statistical software package, version 17.0. The differences in data values were defined as statistically significant at *P* < .05.

## 3 RESULTS

### 3.1 The histopathological characteristics of synovial membranes

**Figure 1:**
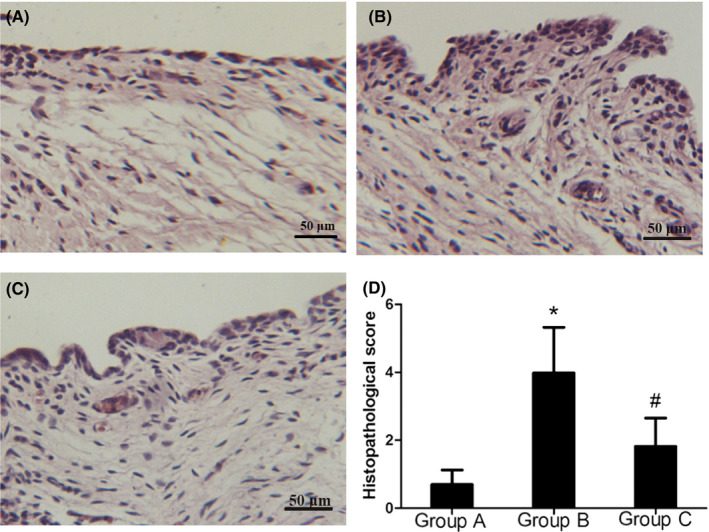
Histopathological changes in the synovium and histopathological scores in the different groups. A, histopathological changes in the synovium of Group A. B, histopathological changes in the synovium of Group B. C, histopathological changes in the synovium of Group C. D, histopathological scores in all the groups. The rat synovium tissues were sectioned in sagittal ffs for HE staining. The severity of inflammation was presented by the histopathological score. The score of Group B was significantly decreased compared to that of Group C. Data associated with all the values were derived from independent samples of n = 6. *P < 0.05 versus Group A, #*P* < 0.05 versus Group B

The results of the histological examination are presented in Figure 1. In Group A (control group), the surface of the synovium was composed of a continuous layer with a thickness of two or three lining cells. There were no obvious inflammatory changes detected in the synovium (Figure 1A). In the synovial membranes of Group B (occlusal interference group), we observed obvious inflammatory changes. The surface layer of the synovium tissue was obviously thickened and less regulated than in Group A. Blood vessel proliferation could also be noticed under the surface layer (Figure 1B). As revealed in Figure 1D, the histopathological score of the synovium in the occlusal interference group was higher than in the control group. In Group C (AMD3100 group), slight hyperplasia of the synovial lining cells and dilated blood vessels were present (Figure 1C). The treatment with AMD3100 significantly reduced the histopathological score in comparison with the scores in the occlusal interference group (Figure 1D).

### 3.2 Expression of SDF‐1 in the TMJ synovium

**Figure 2:**
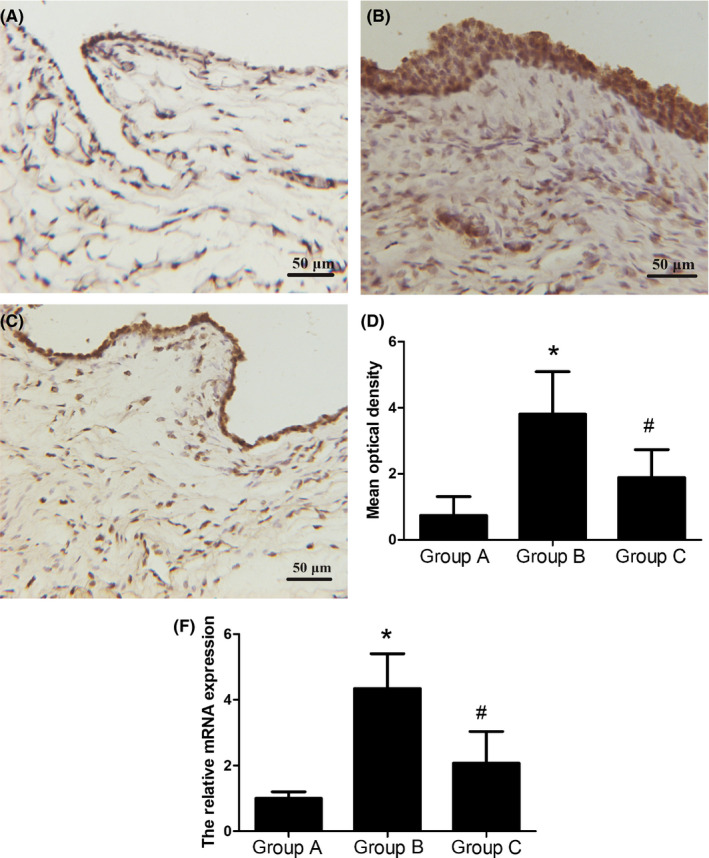
The protein and mRNA expressions of SDF‐1 in the synovium of the TMJ. A, Immunohistochemical staining of SDF‐1 in Group A. B, Immunohistochemical staining of SDF‐1 in Group B. C, Immunohistochemical staining of SDF‐1 in Group C. D, The mean optical density of each of the groups. E, The relative mRNA expression of SDF‐1 in each group. A low expression level could be observed on the surface of the synovial tissue in Group A. The SDF‐1 protein level was significantly higher in Group B comparing to in Group A. Additionally, the surface cells of the synovial tissue were thickened, and a greater number of synoviocytes was stained in Group B. However, the changes were inhibited significantly via treatment with AMD3100. The mRNA expression exhibited the same tendency to change as that observed with the protein results. Data associated with all the values were derived from independent samples of n = 6, *P < 0.05 compared with Group A, #*P* < 0.05 compared with Group B

In order to investigate whether SDF‐1 plays a role in promoting the progression of synovitis, the expressions of the protein of SDF‐1 were measured in each group using immunostaining (Figure 2A‐C). The results revealed a high expression level of the SDF‐1 protein detected in the synoviocytes, especially in the surface layer of the synovial tissue. Compared to in Group A, SDF‐1 was expressed more strongly in Group B. However, the injection with AMD3100 decreased the secretion of SDF‐1 in Group C (Figure 2D). The mRNA expression exhibited the same tendency to change as that observed in the case of the protein results (Figure 2E).

### 3.3 Effect of AMD3100 on the SDF‐1/CXCR4 axis and on the expressions of IL‐1β in the TMJ synovium

**Figure 3:**
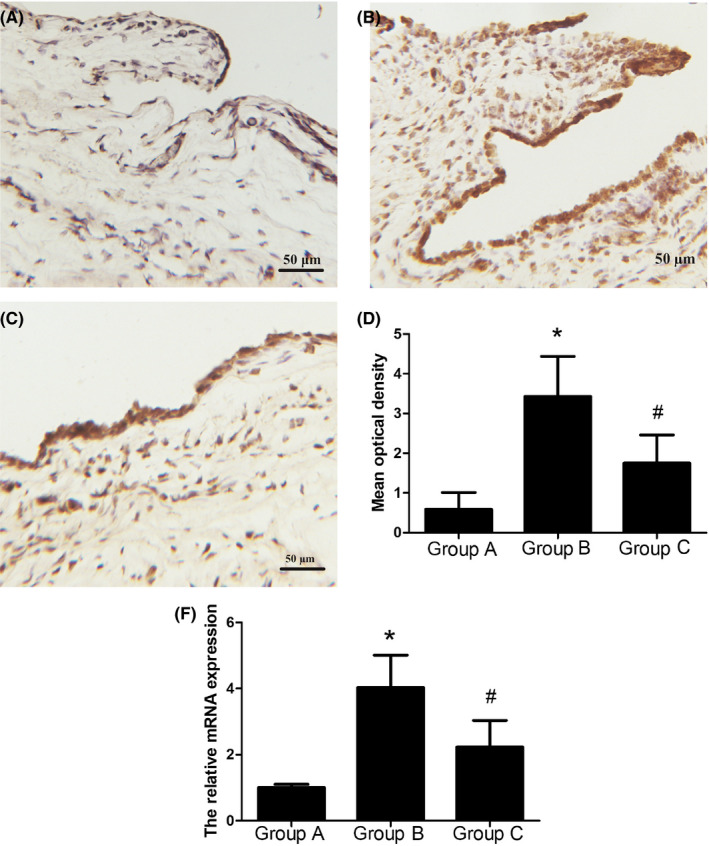
The expressions of IL‐1β in the TMJs in different groups. A, Immunohistochemical staining of IL‐1β in Group A. B, Immunohistochemical staining of IL‐1β in Group B. C, Immunohistochemical staining of IL‐1β in Group C. D, The mean optical density of each of the groups. E, The relative mRNA expression of IL‐1β in each group. The results revealed that IL‐1β was scarcely expressed in the synovial tissue in Group A. Additionally, the expression level of IL‐1β protein in Group B was higher than in Group A. However, in Group C, the IL‐1β secretion level declined significantly compared to in Group B. The mRNA expression exhibited the same tendency to change as that observed with the protein results. Data associated with all the values were derived from independent samples of n = 6, *P < 0.05 compared with Group A, #*P* < 0.05 compared with Group B

In order to detect the expression level of IL‐1β, an immunochemical test was performed. IL‐1β exhibited different shades of staining in the synovial tissue of Groups A, B, and C (Figure 3A‐C). The IL‐1β protein expression increased significantly in Group B compared to in Group A. However, it exhibited a significant decrease owing to the AMD3100 administration, and the MOD in Group C was lower than that in Group B (Figure 3D). To test the expression level of IL‐1β, a real‐time PCR was also performed. The results revealed that the expression of IL‐1β mRNA was significantly higher in Group B compared with in Group A. However, with the injection of AMD3100, the mRNA expressions were remarkably lower in Group B in contrast with in Group C (Figure 3E).

## 4 DISCUSSION

Synovitis is one of the most common diseases affecting the oral and maxillofacial region and is one of the main causes of TMJ pain. The pathogenesis of this condition is not fully understood yet. Additionally, there are many etiologic factors that may be involved in the onset of this disease. Because nociceptors are abundantly present in the synovium and because synovial inflammation increases the responsiveness of the peripheral nociceptive neurons leading to heightened pain sensitivity, the pain caused by synovitis is always intractable.^13^, ^14^ However, there are no safe and valid treatment options to cure this disease. The main treatment option for synovitis is anti‐inflammatory drugs.^15^

In this study, we developed an occlusal interference animal model by performing a technique described in previous experiments involving the bonding of artificial crowns in rats, which simulated the causes of synovitis in the human TMJ to the greatest possible extent.^10^ Some histological changes were found in the TMJ, such as synovial inflammatory changes. Additionally, the surface layer of the synovium tissue in the occlusal interference group was obviously thickened and less regulated than in control group. Further, blood vessel proliferation and fibrin deposition could also be observed under the surface layer. The histopathological score assigned to the synovium in occlusal interference group was higher than in the control group. These findings suggest that occlusal interferences can induce synovial membrane injury in the TMJ. Temporomandibular synovitis occurs due to the TMJ being subjected to excessive force induced by occlusal interferences.^1^

The evidence reveals that the SDF‐1/CXCR4 axis is closely related to various types of joint diseases.^16^, ^17^, ^18^, ^19^ In recent studies, some researchers^16^, ^17^ have tested the levels of SDF‐1 and its association with CXCR4 in the synovium of rheumatoid arthritis patients who were being treated with golimumab, and the results demonstrated that the SDF‐1/CXCR4 axis is closely correlated with disease activity and joint destruction. In this study, the results revealed that in the occlusal interference group, high expression levels of the SDF‐1 protein were detected in the synovial membrane cells, especially in the surface layer of the synovial membrane. Additionally, compared with in the control group, the SDF‐1 expression level was much higher in the occlusal interference group. At the same time, the histopathological score of the synovium was higher in this group as well. This prompted us to consider that SDF‐1 may participate in the regulation of the inflammatory response of the TMJ synovial membranes in rats with occlusal interference.

It has been reported that sustained inflammation is characterized by the up‐regulation of proinflammatory cytokines. IL‐1β could suppress UDP‐glucose dehydrogenase (UGDH)gene expression and consequently inhibit proteoglycans synthesis in articular chondrocytes, which might suppress matrix restoration and contribute to the OA progression.^20^ Many studies have demonstrated that IL‐1β is able to block the chondrocytes from synthesizing extracellular matrix (ECM) components; further, it interferes with the production of key structural proteins, such as type‐II collagen and aggrecan.^21^, ^22^ Furthermore, IL‐1β can also indirectly exert a destructive effect on cartilage components by up‐regulating the synthesis of MMPs, such as MMP‐1, MMP‐3, and MMP‐13.^23^, ^24^ In this study, we observed that IL‐1β was up‐regulated in the occlusal interference group, which corresponds to the histopathological characteristic of the synovial membranes. This finding suggests that IL‐1β plays an important role in sustained inflammation in the TMJ synovium, and sustained inflammation may be a predisposing factor for TMJ degeneration.

It has been demonstrated that in the synovial and articular cartilages, the activation of the SDF‐1/CXCR4 signal pathway can regulate the expression of various inflammatory factors, including IL‐1β, IL‐6, TNF ‐α, and MMPs, which are involved in joint pathology.^2^, ^19^, ^25^, ^26^, ^27^ A previous study has demonstrated that by blocking the SDF‐1/CXCR4 axis with AMD3100, the expressions of IL‐1β and MMP‐13 in chondrocytes harvested from the knee joint of OA patients were significantly reduced.^18^ In our study, compared to in the control group, the SDF‐1 and IL‐1β expression levels and the histopathological score of the synovial membranes were considerably higher in the occlusal interference group. After treatment with AMD3100, all these values exhibited a significant decrease. These findings prompted us to consider that SDF‐1 may play a role in the regulation of the inflammatory response of the TMJ synovial membranes in rats with occlusal interference by regulating the expression levels of inflammatory factors, such as IL‐1β, which can be eliminated by the administration of AMD3100. However, the pathways involved in intracellular signaling transduction between SDF‐1 and the expression of downstream inflammatory mediators, such as IL‐1β, were not studied.

The relationships between TMJ disease and the SDF‐1/CXCR4 axis have rarely been reported on, and the evidence that we uncovered demonstrates that it is closely related to TMJ disease. Our findings suggest that the SDF‐1 axis contributes to synovitis during the development of TMJ disease. Blocking the SDF‐1/CXCR4 signaling pathway by AMD3100 administration may potentially prevent TMD patients from developing synovitis.

## 5 CONCLUSION

In this current study, we found that SDF‐1 regulated the expression levels of inflammatory factors, such as IL‐1β, in the synovial membranes of rats with occlusal interference. Our findings suggest that the SDF‐1 axis may contribute to the onset of synovitis during the development of TMD.

## CONFLICT OF INTERESTS

The authors declare that there are no conflicts of interest associated with the publication of this paper.

## ETHICAL APPROVAL

All the procedures in this study were performed in accordance with the General Recommendation and Provisions of the Chinese Experimental Animals Administration Legislation.

## AUTHOR CONTRIBUTION

**Dong Qi:** Formal analysis. **Shuzhen Sun:** Conceptualization. **Liang Han:** Investigation. **Qiang Wang:** Methodology. **Jingjing Kong:** Supervision. **Yujun Zhang:** Validation. **Ronglin Wang:** Writing‐original draft. **Yan Chen:** Methodology. **Ping Ji:** Data curation.
